# Predicting T-Cell Lymphoma in Children From ^18^F-FDG PET-CT Imaging With Multiple Machine Learning Models

**DOI:** 10.1007/s10278-024-01007-y

**Published:** 2024-02-06

**Authors:** Taiyu Yang, Danyan Liu, Zexu Zhang, Ri Sa, Feng Guan

**Affiliations:** 1 Department of Nuclear Medicine, The First Hospital of Jilin University, 1# Xinmin St, Changchun, 130021 China; 2 Department of Radiology, The First Hospital of Jilin University, 1# Xinmin St, Changchun, 130021 China

**Keywords:** ^18^F-FDG PET/CT, Radiomics, Lymphoma, T-cell, Children

## Abstract

This study aimed to examine the feasibility of utilizing radiomics models derived from ^18^F-FDG PET/CT imaging to screen for T-cell lymphoma in children with lymphoma. All patients had undergone ^18^F-FDG PET/CT scans. Lesions were extracted from PET/CT and randomly divided into training and validation sets. Two different types of models were constructed as follows: features that are extracted from standardized uptake values (SUV)-associated parameters, and CT images were used to build SUV/CT-based model. Features that are derived from PET and CT images were used to build PET/CT-based model. Logistic regression (LR), linear support vector machine, support vector machine with the radial basis function kernel, neural networks, and adaptive boosting were performed as classifiers in each model. In the training sets, 77 patients, and 247 lesions were selected for building the models. In the validation sets, PET/CT-based model demonstrated better performance than that of SUV/CT-based model in the prediction of T-cell lymphoma. LR showed highest accuracy with 0.779 [0.697, 0.860], area under the receiver operating characteristic curve (*AUC*) with 0.863 [0.762, 0.963], and preferable goodness-of-fit in PET/CT-based model at the patient level. LR also showed best performance with accuracy of 0.838 [0.741, 0.936], AUC of 0.907 [0.839, 0.976], and preferable goodness-of-fit in PET/CT-based model at the lesion level. ^18^F-FDG PET/CT-based radiomics models with different machine learning classifiers were able to screen T-cell lymphoma in children with high accuracy, *AUC*, and preferable goodness-of-fit, providing incremental value compared with SUV-associated features.

**Supplementary Information:**

The online version contains supplementary material available at 10.1007/s10278-024-01007-y.

## Introduction

Lymphoma, a malignant tumor that originates from lymph nodes and/or extranodal lymphoid tissue, is the third most common malignancy in children (0–14 years old) [1]. It can be further classified as Hodgkin lymphoma (HL) and non-Hodgkin lymphoma (NHL). HL derives from a specific abnormal B lymphocyte lineage, while NHL may originate from either abnormal B or T cells. The therapeutic outcomes and prognosis differ widely between B-cell and T-cell lymphoma. Children with B-cell lymphoma have a multitude of options for therapy from chemotherapy to targeted antibodies. Most children in this entity generally have excellent event-free survival and overall survival following comprehensive treatment for primary disease [2–5]. Compared with B-cell lymphoma, even though T-cell lymphomas can have favorable response rates to initial chemotherapy, relapse and refractory disease are common and can be more difficult to treat due to the intrinsic biological characteristics such as the potential toxicity of T-cell aplasia and the complexities of fratricide [6, 7]. In addition, the rarity of T-cell lymphomas can make it more difficult to develop effective treatment approaches, as there are fewer patients available for clinical trials and research studies [8]. This can lead to a lack of consensus on the best treatment approach for T-cell lymphomas.

The cell origin of lymphoma has been conventionally evaluated based on biopsy, which involves the removal of a tissue sample from a lymph node, bone marrow, or other affected area [9]. Biopsy is generally a safe procedure, but like any invasive medical procedure, it carries some risks and potential side effects, including bleeding, pain, infection, and damage to nearby organs or structures, especially in children [10]. Compared to adults, children may be more susceptible to certain risks of biopsy due to their smaller size and developing bodies. For example, the risk of bleeding during a biopsy may be higher in children, especially in infants or very young children because of their smaller blood vessels [11]. Another consideration is the fact that children may be more anxious or scared during the procedure, which can make it more difficult to perform the biopsy and may increase the risk of complications. In some cases, the location of the biopsy may also affect the risks involved. For example, if a biopsy is performed on the bone, there is a risk of damage to the growth plate, which can affect the child’s bone growth. If a biopsy is performed on the brain, there is a risk of neurological complications such as seizures or bleeding [12]. Thus, it can be a stressful experience for both the child and their parents.

Certain imaging features may be associated with specific tumor types or biological characteristics, which may be a substitute for biopsy. Recently, 2-[^18^F] fluoro-2-deoxy-D-glucose positron emission tomography/computed tomography (^18^F-FDG PET/CT) is broadly performed before, during, after treatment of lymphoma for accurate staging, treatment planning, and response assessment [13, 14]. It is not affected by sampling position and sampling size. It can discover almost all invaded lymph nodes and extranodal organs with one scan in lymphoma, including lymph nodes smaller than 1 cm with high uptake of ^18^F-FDG. A few earlier studies have summarized the ^18^F-FDG PET/CT imaging performance of different kinds of lymphoma [15, 16]. However, the analyses in these studies were largely based on several semi-quantitative parameters of standardized uptake values (SUV) such as SUV_max_, SUV_peak_, and SUV_mean_. These parameters are measured on a single or partial voxel within the volume of interest (VOI), lacking consideration of the spatial relationship between individual voxels [17, 18]. In addition, these studies mainly emphasized the impact of PET/CT on adult lymphoma, while only several studies have emerged to evaluate the utility of PET/CT in young children [19, 20]. Thus, it is necessary to develop complementary approaches to dig out the entire parameters of pretreatment ^18^F-FDG PET/CT to reflect the underlying information of T-cell lymphoma in children.

Radiomics is an emerging non-invasive method that extracts high-throughput data, called radiomic features, from medical images and then uses machine learning methods to perform quantitative analysis and prediction of diseases. Compared with traditional methods, radiomics can be conceived as digital biopsy, which predicts pathological results through image data [21, 22]. With the development of computer science and artificial intelligence technology, the field of radiomics is growing exponentially and has been employed for the differential diagnosis of many diseases [23, 24]. The second- or higher-order features extracted by radiomics can characterize the tumor microenvironment and tumor phenotype, which are different from the first-order histogram [25, 26]. Several studies have demonstrated that ^18^F-FDG PET/CT-based radiomics approaches can distinguish lymphoma from other primary malignancies [27, 28]. Even more, ^18^F-FDG PET/CT-based radiomics can successfully differentiate follicular lymphoma from diffuse large B lymphoma [29]. Given this, we conducted this study to determine whether radiomics models based on clinical baseline ^18^F-FDG PET-CT functional imaging with different machine learning classifiers can be used to screen T-cell lymphoma in children before biopsy. Furthermore, we also aimed to compare the performance of SUV/CT-based model and PET/CT-based model in the prediction of cell origin in lymphoma.

## Methods

### Patient Population

This retrospective study was permitted by the Ethical Committee of the First Hospital of Jilin University (2023–299). This study selected a cohort from the medical record database from our institute from February 2017 to July 2022. The inclusion criteria included having: (i) age ≤ 14 years old; (ii) patients who underwent ^18^F-FDG PET/CT with good image quality of 4 points or 5 points by using a 5-point Likert scale [30, 31]; (iii) patients who underwent histological biopsy within 6 weeks before or after ^18^F-FDG PET/CT, and histologically confirmed as lymphoma. The exclusion criteria consisted of having: (i) patients who had received chemotherapy, surgery, or other treatments before PET/CT; (ii) pathology that could not confirm whether the lymphoma was T-cell originated or non-T-cell originated; (iii) no clear lesion on the PET/CT image or the lesion that could not be accurately segmented (e.g., adjacent to areas of high physiologic uptake).

### ^*18*^*F-FDG PET/CT Acquisition*

^18^F-FDG PET/CT scans were performed using a Siemens Biograph 16 HR PET/CT according to the European Association of Nuclear Medicine (EANM) procedure guidelines for tumor imaging [32]. The radioactive tracer ^18^F-FDG was automatically synthesized by the cyclotron (Sumitomo, Japan), and the radiochemical purity was higher than 95%. Patients should fast for more than 6 h before the examination and ensure that the glucose level is less than 11.1 mmol/L. Inject ^18^F-FDG imaging agent based on patient body weight (dose of 3 MBq per kg body weight), and PET/CT image acquisition was started after 60 min of rest.

The patient first underwent a whole-body low-dose CT scan in the head first-supine position, followed by a whole-body ^18^F-FDG PET scan with 100 s per bed position. PET data were reconstructed using the ordered subset expectation maximization (OSEM, 24 subsets, and 2 iterations), and CT information was used for attenuation correction and scatter correction.

### Lesion Segmentation

The PET/CT images acquired from the patients were imported into the 3D Slicer (Version 5.0.2, www.slicer.org) software, and the lesions were segmented using the “segment editor” module of the software. Lesion segmentation was performed at the patient and lesion levels.

At the patient level, according to previous studies, a semi-automatic segmentation method was used to delineate ^18^F-FDG-avid lesions with a SUV_max_ of at least 4.0 on PET images by 3D Slicer as a VOI for each patient [33]. Regions were removed if they were interpreted as physiological metabolic uptake or extranoal involvement. For some lesions that were not automatically segmented but suspected to be malignant, the margins of the VOI were manually adjusted.

**Fig. 1:**
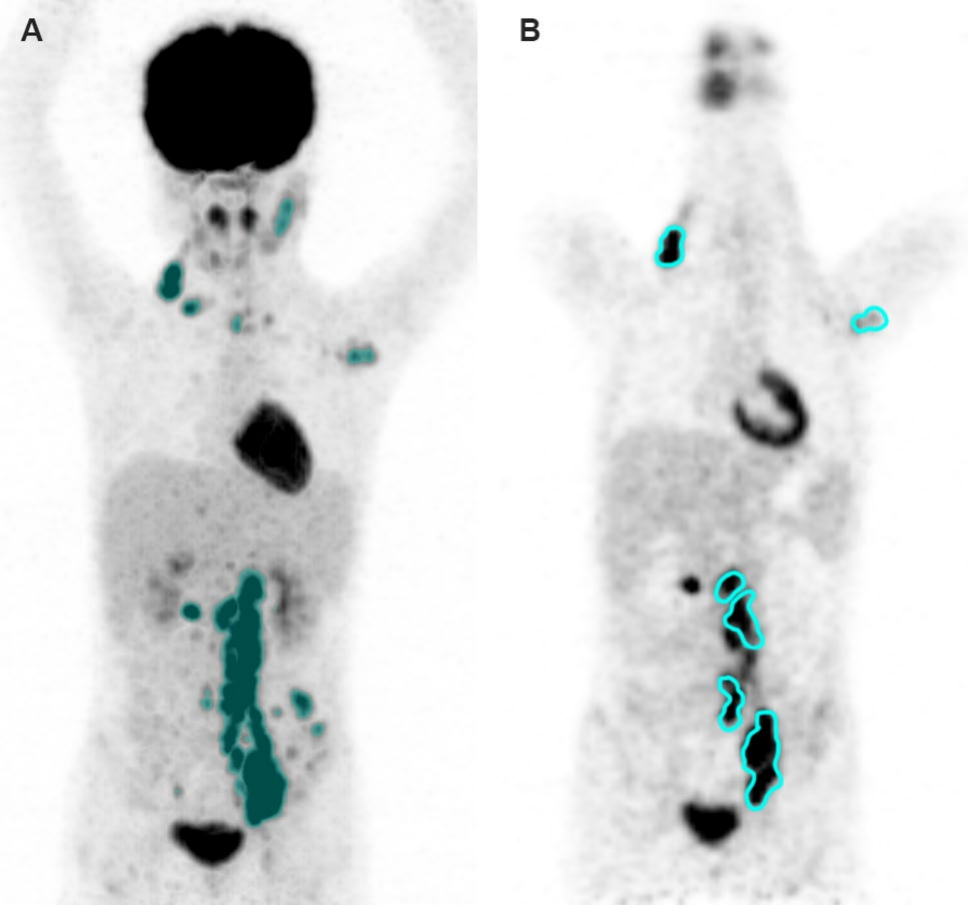
Example of lesion segmentation. **A** Maximum intensity projection for lesion segmentation at the patient level; **B** PET for lesion segmentation at the lesion level

At the lesion level, lesions were selected in terms of SUV_max_ level, and lymph node distribution was done manually. We segmented at most ten VOI for each patient. If multiple lesions were distributed in different regions (≥ 2) such as neck, clavicle, mediastinum, and abdominal cavity, at least one lymph node with highest SUV_max_ in different regions were selected. If multiple lesions were gathered in one region, at least two lesions with the highest SUV_max_ were selected. Ultimately, two experienced nuclear medicine physicians reviewed the selected VOIs at both levels. An example of lesion segmentation is shown in Fig. 1.

### Feature Extraction and Selection

Feature extraction and selection were performed in Python 3.9.12 using the open-source libraries PyRadiomics [34] (v3.0.1) and scikit-learn [35] (v1.0.2), respectively. First, 14 shape features were extracted for each VOI, and then the following radiomics features were extracted from PET and CT images, respectively: 18 first-order statistical features, 24 gray level co-occurrence matrix (GLCM) features, 14 gray level dependence matrix (GLDM) features, 16 gray level run length matrix (GLRLM) features, 16 gray level size zone features matrix (GLSZM) features, 5 neighboring gray tone difference matrix (NGTDM) features, and 744 wavelet features. In addition, 22 SUV associated semi-quantitative indicators such as SUV_max_, SUV_peak_, and SUV_mean_ for each VOI were calculated using the PET-IndiC plugin of 3D Slicer.

Levene’s test was used to assess the equality of variances for all 1710 features to make the decision on whether to use an independent two-sample *t*-test or the Welch’s *t*-test [36]. Then, features with statistical significance (*P* < 0.05) were kept in the subset of radiomics features. Furthermore, the features were dimensionality reduced using least absolute shrinkage and selection operator (LASSO) regression through 100,000 iterations and 10 cross-validations, and the dimensionality-reduced features were used to build radiomics models.

### Model Development and Validation

The machine learning models were built and trained on a per patient level and a per lesion level, respectively. Firstly, all patients or lesions were randomly divided into training and validation sets with a ratio of 7:3 by 10 times cross-validation. Then, two different types of models were built. The first model was named as PET/CT-based model. In this model, CT radiomics and PET radiomics features were analyzed. The second model was named as SUV/CT-based model. In this model, CT radiomics features and only SUV-associated semi-quantitative indicators extracted from PET were analyzed. Subsequently, five different machine learning classifiers including logistic regression (LR), linear support vector machine (LSVM), support vector machine with the radial basis function kernel (SVM-RBF), neural networks (NN), and adaptive boosting (ADA) were applied to each constructed model.

In order to evaluate the impact of each feature on the model’s prediction results, we used SHapley Additive exPlanations (SHAP) summary plot to evaluate the contribution that 10 most influential features made to a predicted value in each model with different machine learning classifiers. “0” was considered as non-T-cell lymphoma, and “1” was defined as T-cell lymphoma in SHAP summary plot. Positive SHAP values increase the likelihood of prediction of T-cell lymphoma, whereas negative SHAP values decrease the likelihood of prediction of T-cell lymphoma.

**Fig. 2:**
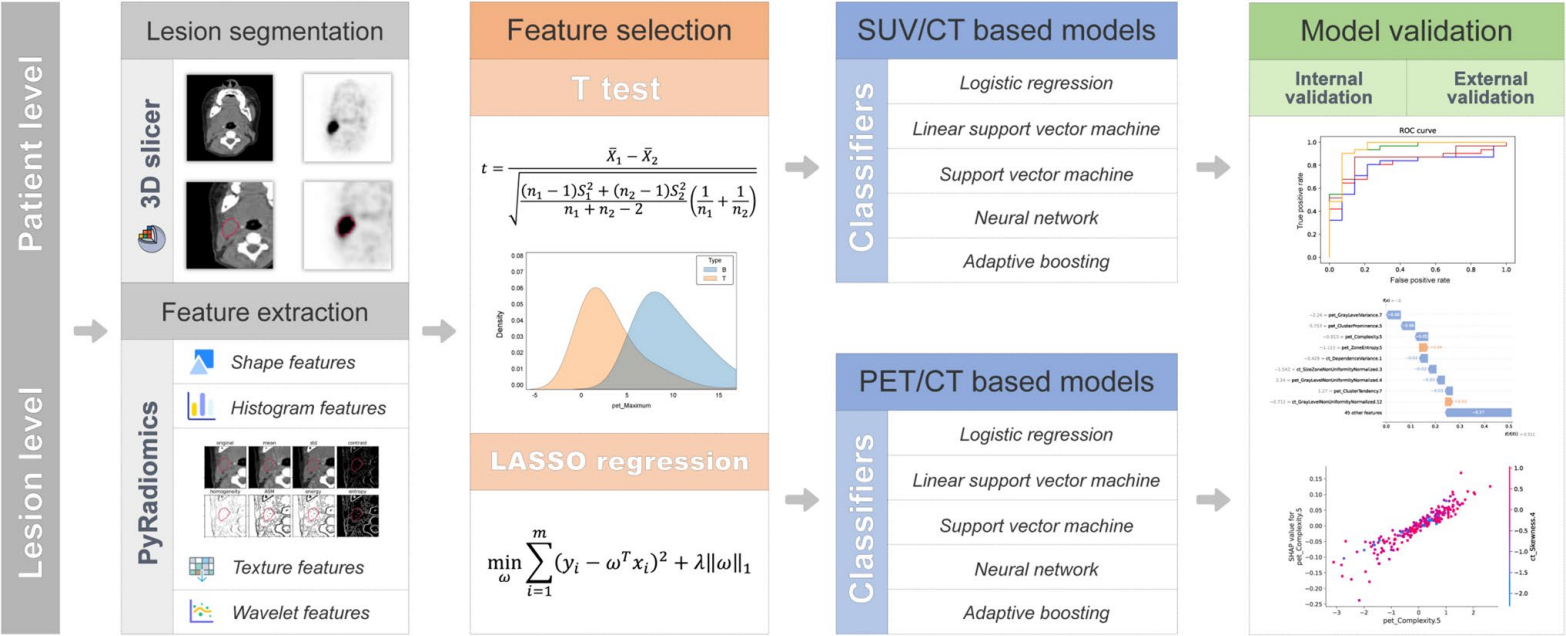
The radiomics based machine learning workflow for screening T-cell lymphoma from in children

Receiver operating characteristic (ROC) curves were drawn to quantify the sensitivity, specificity, and accuracy of each model in the validation set. The corresponding values of the area under the ROC curve (*AUC*) were used to evaluate the diagnostic performance of each model. Hosmer–Lemeshow (HL) tests and calibration curves were performed to assess the goodness-of-fit. Delong test was used to compare the statistical difference of *AUC* between PET/CT-based models and SUV/CT-based models. Figure 2 shows the radiomics analysis pipeline.

## Results

### Demographic Characteristics

We collected information of 142 children with lymphoma who underwent ^18^F-FDG PET/CT in our institute. Among them, we excluded 7 patients who had received other treatments, 20 patients whose pathological diagnosis was not confirmed whether the lymphoma was T-cell-originated or not, and 5 patients with no clear lymph node lesions on PET/CT images. Finally, a total of 110 lymphomas including 26 T-lymphoblastic leukemia/lymphomas (T-ALL/T-LBL), 10 anaplastic large cell lymphomas (ALCL), 15 unclassified T-cell lymphomas, 14 B lymphoblastic leukemia/lymphomas (B-ALL/B-LBL), 11 diffuse large B-cell lymphomas (DLBCL), 14 Hodgkin’s lymphomas (HL), 16 Burkitt lymphomas, and 4 unclassified B-cell lymphoma patients were enrolled. The basic information of the patients is shown in Table 1.

**Table 1:** Basic information for patients

	**All patients (*n* = 110)**	**T cell lymphoma (*n* = 51)**	**Non-T cell lymphoma (*n* = 59)**	***P*-value**
Gender				0.031*
Male	74 (67%)	29 (57%)	45 (76%)	
Female	36 (33%)	22 (43%)	14 (24%)	
Age (years)	8.6 ± 3.60	8.6 ± 3.70	8.4 ± 3.60	0.774
Ann arbor staging				0.069
I–II	26 (24%)	8 (16%)	18 (30%)	
III–IV	84 (76%)	43 (84%)	41 (70%)	
Elevated LDH				0.184
Yes	48 (44%)	19 (37%)	29 (49%)	
No	59 (54%)	31 (61%)	28 (48%)	
NA	3 (2%)	1 (2%)	2 (3%)	
ECOG score				0.381
0–1	98 (89%)	44 (86%)	54 (92%)	
≥ 2	12 (11%)	7 (14%)	5 (8%)	
Extranodal infiltration				0.184
0	59 (54%)	31 (61%)	28 (48%)	
≥ 1	48 (44%)	19 (37%)	29 (49%)	
NA	3 (2%)	1 (2%)	2 (3%)	
IPI score				0.976
0	4 (4%)	2 (4%)	2 (3%)	
1–2	57 (52%)	27 (53%)	30 (51%)	
3–5	46 (42%)	21 (41%)	25 (43%)	
NA	3 (3%)	1 (2%)	2 (3%)	

*LDH* lactate dehydrogenase, *ECOG* Eastern Cooperative Oncology Group performance status, *IPI* international prognostic index

^*^P < 0.05; **P < 0.01

### Feature Extraction and Selection

We extracted 1710 radiomics features at the patient level and the lesion level, respectively. At the patient level, a total of 51 T-cell lymphoma patients and 59 non-T-cell lymphoma patients were enrolled. After applying an independent two-sample *t*-test, we discarded 1524 invalid features and retained 186 features which showed statistical significance (*P* < 0.05). After LASSO regression dimensionality reduction, 16 features of PET/CT-based model and 10 features of SUV/CT-based model were retained for building the final models, respectively. At the lesion level, a total of 163 T-cell lymphoma lesions and 191 non-T-cell lymphoma lesions were segmented. After applying an independent two-sample *t*-test, 694 features with statistical significance (*P* < 0.05) were retained. After LASSO regression dimensionality reduction, 54 features of PET/CT-based model and 34 features of SUV/CT-based model were retained for building the final models, respectively. LASSO regression for feature selection is shown in Supplementary Fig. 1. The correlation matrix of the retained features is presented in Supplementary Fig. 2.

### Model Development and Validation

There were 37 T-cell lymphoma patients (23 T-ALL/T-LBL, 6 ALCL, 8 unclassified T-cell lymphoma), and 40 non-T-cell lymphoma patients (9 B-ALL/B-LBL, 8 DLBCL, 10 HL, 10 Burkitt lymphoma, and 3 unclassified B-cell lymphoma) in the training set, while 14 T-cell lymphoma patients (3 T-ALL/T-LBL, 4 ALCL, and 7 unclassified T-cell lymphoma), and 19 non-T-cell lymphoma patients (5 B-ALL/B-LBL, 3 DLBCL, 4 HL, 6 Burkitt lymphoma, and 1 unclassified B-cell lymphoma) in the validation set at the patient level. There were 107 T-cell lymphoma lesions and 140 non-T-cell lymphoma lesions in the training set, while 56 T-cell lesions and 51 non-T-cell lesions in the validation set at the lesion level. The information of VOI is given in Supplementary Table 1.

**Fig. 3:**
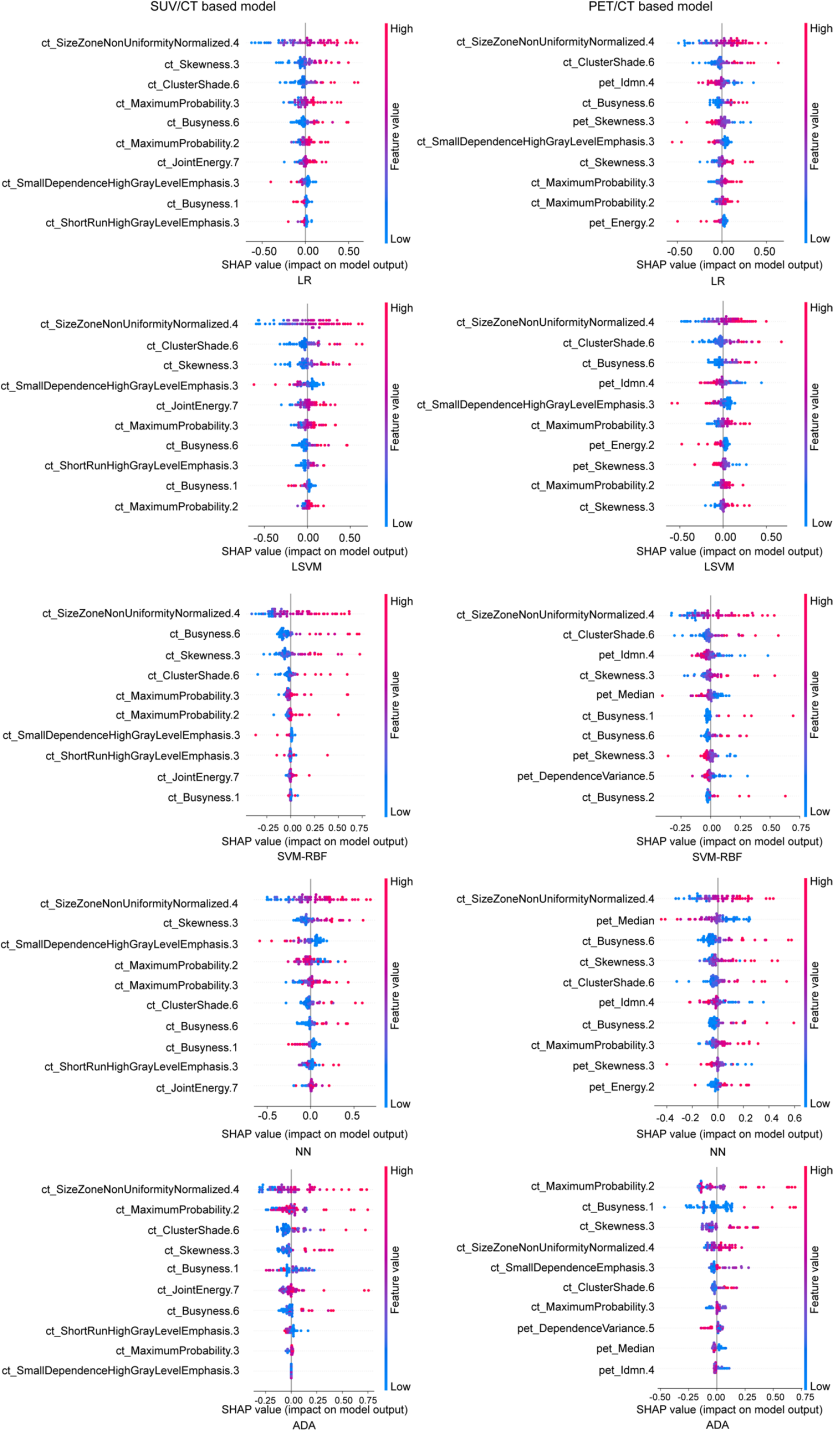
SHapley Additive exPlanations summary plot for the evaluation for the contribution that 10 most influential features made to a predicted value in SUV/CT-based model and PET/CT-based model with logistic regression, linear support vector machine, support vector machine with the radial basis function kernel, neural networks, and adaptive boosting at the patient level

**Fig. 4:**
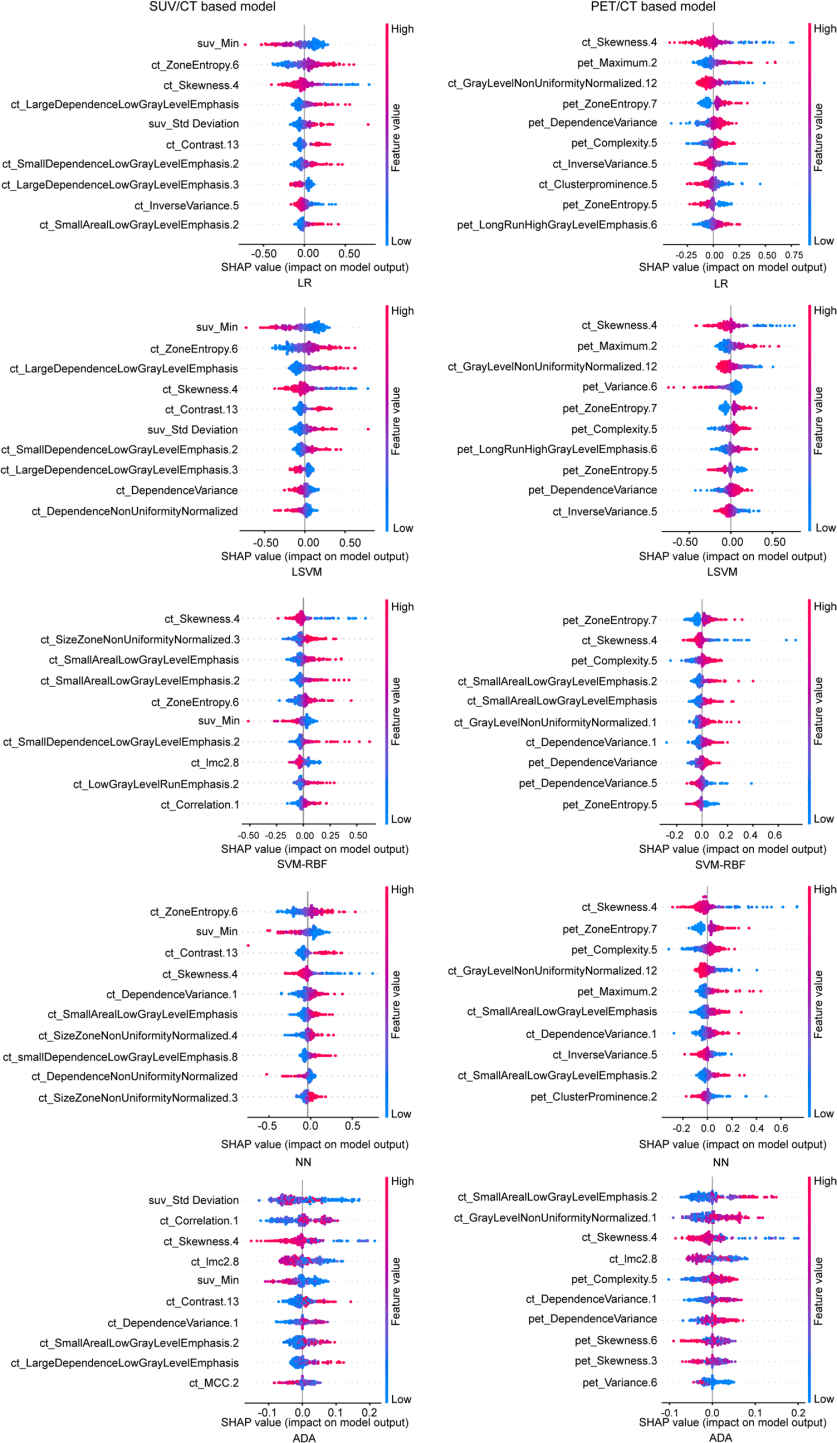
SHapley Additive exPlanations summary plot for the evaluation for the contribution that 10 most influential features made to a predicted value in SUV/CT-based model and PET/CT-based model with logistic regression, linear support vector machine with the radial basis function kernel, support vector machine, neural networks, and adaptive boosting at the lesion level

The top 10 important features of each model with different classifiers were visualized by SHAP in the training set at the patient level. In SUV/CT-based models, it was found that the top 10 most influential features originated solely from CT, whereas the features from SUV did not demonstrate significant importance. The PET/CT-based models reveal that the influential features ranking among the top 10 influential features for each type of classifier are derived from both PET and CT imaging techniques, as shown in Fig. 3. The relationship between the top 10 important features of each model with different classifiers was visualized by SHAP in the training set at the lesion level. In SUV/CT-based models, the majority of influential features were originated from CT, whereas only one or two features were originated from SUV. Conversely, in PET/CT-based models, most of the contributed features are from PET, while ct_GrayLevelNonUniformityNormalized.12 from CT is consistently among the top 10 influential features for each type of classifier, as shown in Fig. 4.

**Fig. 5:**
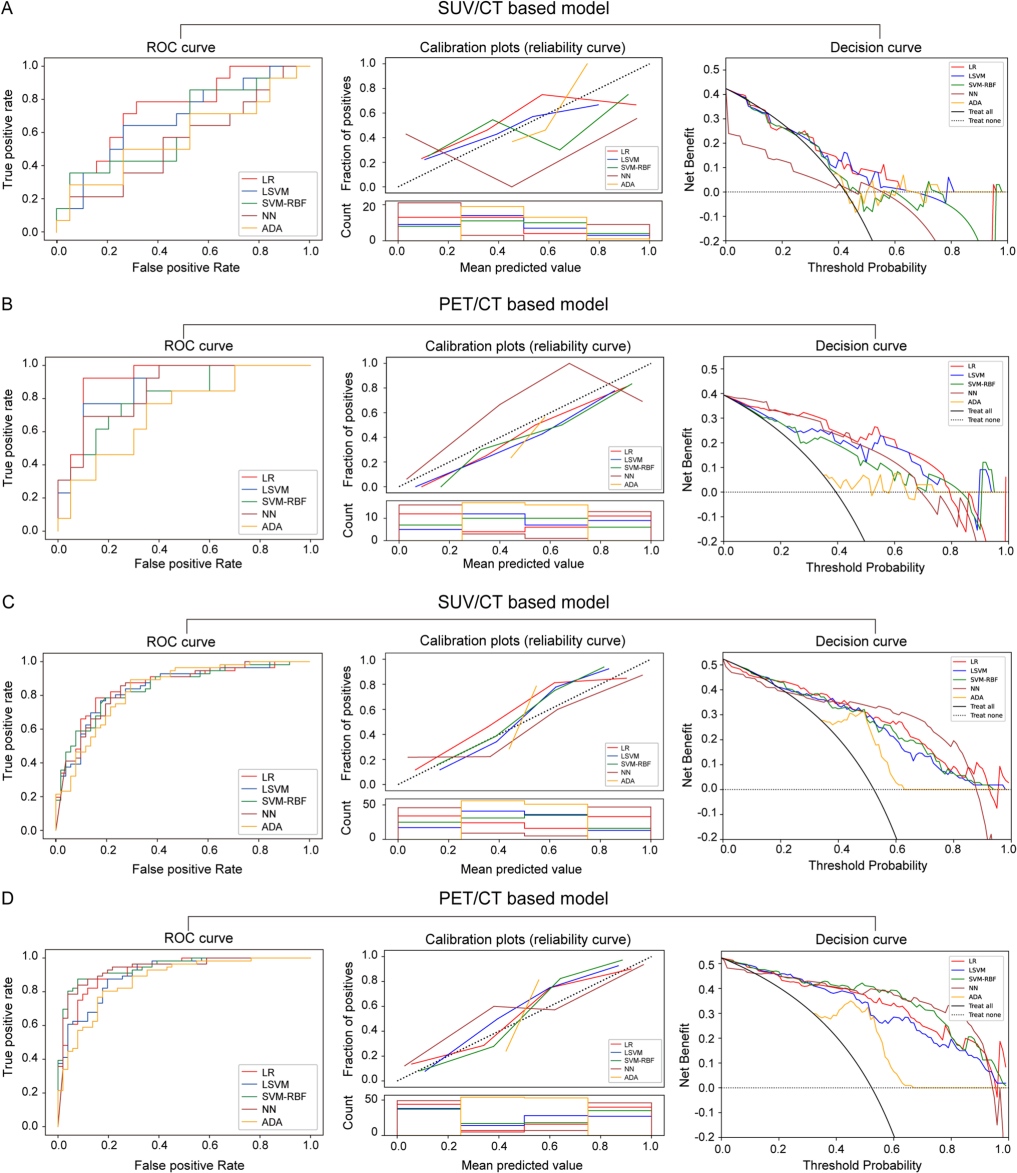
Comparison of receiver operating characteristic (ROC) curves, Hosmer–Lemeshow test and decision curve analysis for predicting T-cell lymphoma in SUV/CT-based model and PET/CT-based model with logistic regression, linear support vector machine, support vector machine with the radial basis function kernel, neural networks, and adaptive boosting. **A** ROC, calibration curves and decision curve analysis in SUV/CT-based model at the patient level; **B** ROC, calibration curves, and decision curve analysis in PET/CT-based model at the patient level; **C** ROC, calibration curves and decision curve analysis in SUV/CT-based model at the lesion level; **D** ROC, calibration curves and decision curve analysis in PET/CT-based model at the lesion level

The sensitivity, specificity, accuracy, and *AUC* of each model with different machine learning classifiers in the validation set at the patient level are shown in Table 2. In SUV/CT-based model, the LR classifier obtains the highest accuracy of 0.727 [0.630, 0.825] and an *AUC* of 0.804 [0.714, 0.893], and the HL test shows preferable goodness-of-fit, and most of the decision curve analyses (DCA) show good performance, as shown in Fig. 5A. In PET/CT-based model, the LR classifier also achieves the highest accuracy of 0.779 [0.697, 0.860] and *AUC* of 0.863 [0.762, 0.963], and the HL test shows preferable goodness-of-fit, and the DCA shows good performance, as shown in Fig. 5B. The sensitivity, specificity, accuracy, and *AUC* of each model with different machine learning classifiers in the validation set at the lesion level are shown in Table 3. In SUV/CT-based model, the LR classifier obtained the highest accuracy of 0.730 [0.675, 0.785] and an *AUC* of 0.817 [0.753, 0.880], but the HL test showed poor goodness-of-fit. LSVM classifier ranks second with an accuracy of 0.723 [0.663, 0.784] and *AUC* of 0.811 [0.746, 0.876], and the HL test shows preferable goodness-of-fit, and the DCA shows good performance, as shown in Fig. 5C. In PET/CT-based model, the NN classifier achieved the highest accuracy of 0.852 [0.752, 0.953] and *AUC* of 0.923 [0.860, 0.987], but the HL test showed poor goodness-of-fit. LR classifier ranked second with an accuracy of 0.838 [0.741, 0.936] and *AUC* of 0.907 [0.839, 0.976], and the HL test shows preferable goodness-of-fit, and the DCA shows good performance, as shown in Fig. 5D. The diagnostic performance of LR, LSVM, SVM-RBF, and NN showed no significant difference in Delong test at both patient and lesion levels.

**Table 2:** Sensitivity, specificity, accuracy, and *AUC* of each model at the patient level in the validation set

	**Sensitivity [95% *CI*]**	**Specificity [95% *CI*]**	**Accuracy [95% *CI*]**	***AUC* [95% *CI*]**	***P*-value**
SUV/CT-based model					
LR	0.615 [0.392, 0.838]	0.823 [0.638, 1.008]	0.727 [0.630, 0.825]	0.804 [0.714, 0.893]	0.018*
LSVM	0.562 [0.410, 0.715]	0.795 [0.601, 0.989]	0.688 [0.586, 0.789]	0.785 [0.678, 0.891]	0.074
SVM-RBF	0.532 [0.337, 0.727]	0.837 [0.589, 1.085]	0.694 [0.574, 0.813]	0.752 [0.625, 0.878]	0.202
NN	0.513 [0.256, 0.770]	0.718 [0.523, 0.913]	0.624 [0.515, 0.733]	0.693 [0.547, 0.839]	0.444
ADA	0.566 [0.348, 0.785]	0.703 [0.464, 0.941]	0.645 [0.551, 0.740]	0.683 [0.530, 0.836]	0.466
PET/CT-based model					
LR	0.744 [0.584, 0.904]	0.806 [0.649, 0.964]	0.779 [0.697, 0.860]	0.863 [0.762, 0.963]	< 0.001**
LSVM	0.707 [0.538, 0.876]	0.781 [0.594, 0.967]	0.745 [0.655, 0.836]	0.837 [0.719, 0.955]	< 0.001**
SVM-RBF	0.674 [0.498, 0.851]	0.775 [0.511, 1.000]	0.724 [0.565, 0.883]	0.823 [0.655, 0.990]	0.003**
NN	0.717 [0.601, 0.832]	0.731 [0.530, 0.931]	0.721 [0.604, 0.839]	0.810 [0.703, 0.917]	< 0.001**
ADA	0.606 [0.376, 0.836]	0.738 [0.504, 0.971]	0.676 [0.509, 0.843]	0.733 [0.543, 0.922]	0.033*

*AUC* area under the receiver operating characteristic curve, *CI* confidence interval, *LR* logistic regression, *LSVM* linear support vector machine, *SVM-RBF* support vector machine with the radial basis function kernel, *NN* neural network, *ADA* adaptive boosting

^*^P < 0.05; **P < 0.01

**Table 3:** Sensitivity, specificity, accuracy, and *AUC* of each model at the lesion level in the validation set

	**Sensitivity [95% *CI*]**	**Specificity [95% *CI*]**	**Accuracy [95% *CI*]**	***AUC* [95% *CI*]**	***P*-value**
SUV/CT-based model					
LR	0.744 [0.631, 0.858]	0.719 [0.653, 0.785]	0.730 [0.675, 0.785]	0.817 [0.753, 0.880]	< 0.001**
LSVM	0.740 [0.635, 0.846]	0.711 [0.642, 0.779]	0.723 [0.663, 0.784]	0.811 [0.746, 0.876]	< 0.001**
SVM-RBF	0.688 [0.561, 0.815]	0.741 [0.645, 0.836]	0.715 [0.664, 0.766]	0.804 [0.730, 0.878]	< 0.001**
NN	0.731 [0.623, 0.840]	0.715 [0.601, 0.829]	0.721 [0.641, 0.802]	0.805 [0.752, 0.857]	< 0.001**
ADA	0.715 [0.586, 0.844]	0.720 [0.614, 0.826]	0.717 [0.652, 0.782]	0.768 [0.701, 0.835]	< 0.001**
PET/CT-based model					
LR	0.827 [0.682, 0.973]	0.847 [0.748, 0.945]	0.838 [0.741, 0.936]	0.907 [0.839, 0.976]	< 0.001**
LSVM	0.809 [0.656, 0.962]	0.831 [0.733, 0.930]	0.821 [0.720, 0.923]	0.895 [0.810, 0.979]	< 0.001**
SVM-RBF	0.757 [0.606, 0.908]	0.854 [0.756, 0.952]	0.808 [0.724, 0.893]	0.906 [0.837, 0.974]	< 0.001**
NN	0.842 [0.729, 0.954]	0.862 [0.744, 0.981]	0.852 [0.752, 0.953]	0.923 [0.860, 0.987]	< 0.001**
ADA	0.758 [0.651, 0.865]	0.753 [0.608, 0.898]	0.754 [0.697, 0.812]	0.830 [0.763, 0.898]	< 0.001**

*AUC* area under the receiver operating characteristic curve, *CI* confidence interval, *LR* logistic regression, *LSVM* linear support vector machine, *SVM-RBF* support vector machine with the radial basis function kernel, *NN* neural network, *ADA* adaptive boosting

^*^P < 0.05; **P < 0.01

**Fig. 6:**
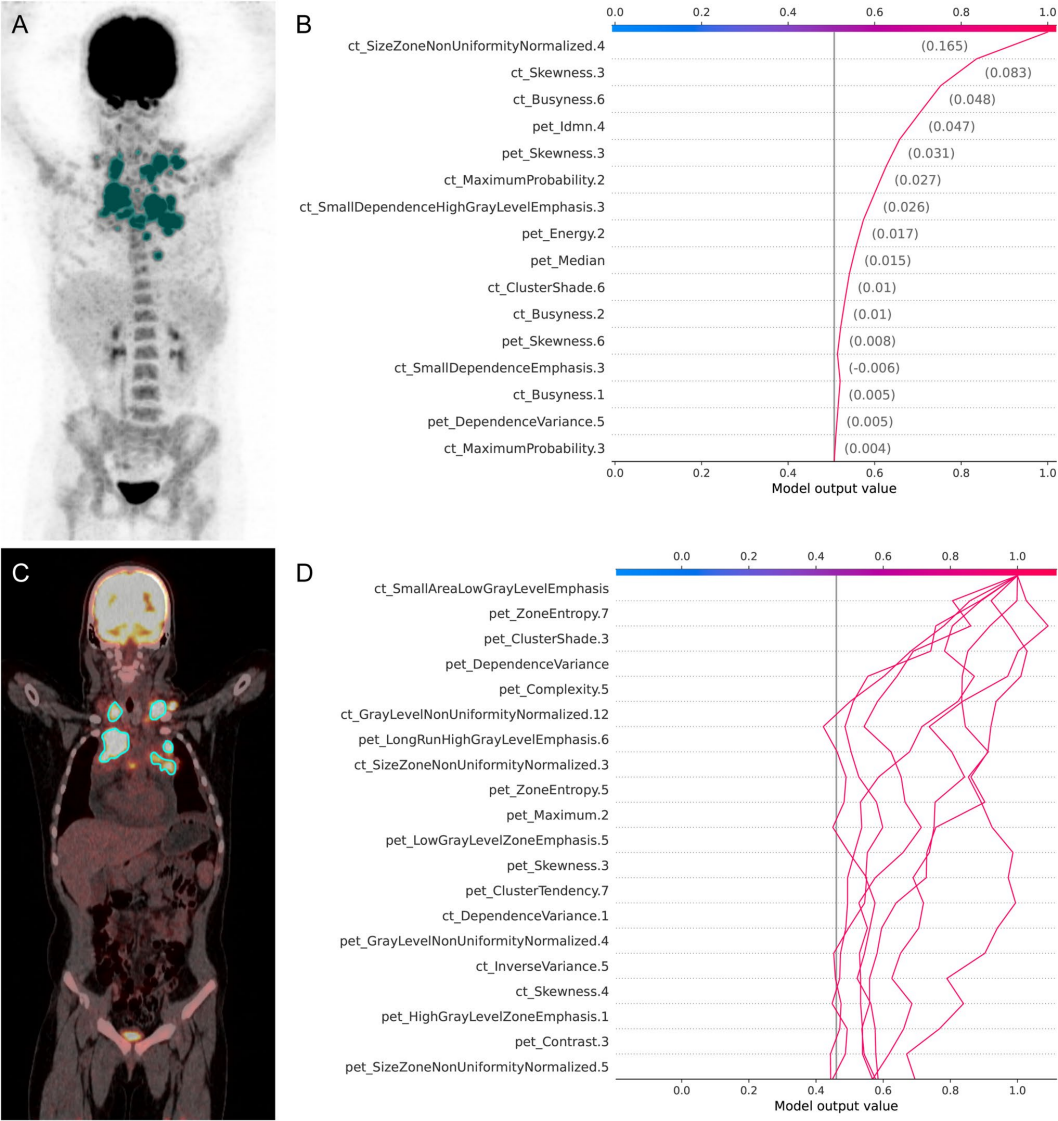
Using PET/CT based model with Logistic Regression classifier to predict cell origin in a 9-year-old male child, who was pathologically confirmed as T-cell lymphoma. **A** Maximum intensity projection for delineation of segmented lesions at patient level; **B** SHAP decision plots showing prediction of T-cell lymphoma at the patient level; **C** Fused PET/CT for delineation of segmented lesions at the lesion level; **D** SHAP decision plots showing prediction of T-cell lymphoma at the lesion level

The representative case with prediction of T-cell lymphoma from ^18^F-FDG PET/CT imaging at the patient and lesion levels are shown in Fig. 6.

## Discussion

To the best of our knowledge, this is the first study to report on the application of radiomics model based on ^18^F-FDG PET-CT imaging to screen for T-cell lymphoma in children. In this study, we developed and validated SUV/CT- and PET/CT-based machine learning model and compared the diagnostic performance of different machine learning classifiers at both patient and lesion levels.

Currently, there is no consensus on the best segmentation method for radiomics of lymphoma lesions, as the disease often involves many different lymph nodes and extranodal organs throughout the body. Due to the high inter- and intra-tumoral heterogeneity within patients, the patient level VOI best reflects the disease burden. Therefore, some studies calculated radiomics features at the patient level. However, as texture features become difficult to interpret at the patient level, other studies only delineated VOIs at the lesion level [37]. In addition, according to the study by Eertink et al., the patient level radiomics features are less affected by the segmentation method compared to the lesion level, so our study used different segmentation methods for the two levels [33]. The PET/CT-based model with LR had the best performance at both patient and lesion levels.

Both SUV/CT-based and PET/CT-based machine learning analysis take advantage of the complementary information provided by different imaging modalities, potentially providing more accurate and comprehensive information about the biology and behavior of tumors [22, 26]. But they focus on different types of quantitative features. SUV/CT-based machine learning analysis concentrates specifically on the SUV values measured within the PET images, along with other quantitative features extracted from the CT images. Whereas, PET/CT-based machine learning involves analyzing a variety of quantitative features extracted from both the PET and CT images, including parameters such as texture, shape, and intensity. PET/CT-based machine learning analysis may be more comprehensive and provide more information about the biology of tumors, but it requires more complex and time-consuming image processing. SUV/CT-based machine learning analysis may be simpler and more straightforward, but it relies more heavily on the accuracy and reproducibility of the SUV measurements [17]. The study found that the *AUC* and accuracy of predicting T-cell lymphoma in PET/CT-based machine learning analysis were higher than those of SUV/CT-based machine learning analysis, suggesting that PET/CT-based machine learning may be more suitable for predicting T-cell lymphoma in children lymphoma.

Many machine learning algorithms suffer from “epistemic opacity,” and the interpretability of machine learning models largely hinders the general application of radiomics models [38]. To ensure the broad applicability, we employed five different machine learning classifiers including LR, LSVM, SVM-RBF, NN, and ADA for analysis. In our study, the LR classifier exhibited the best predictive capability at both patient and lesion levels testing cohort. LR is a widely used classification algorithm, which assumes that the data follows a certain probability distribution, and estimates the parameters by maximizing the likelihood function. It is known for its simplicity, easy implementation, high computational efficiency, and good interpretability, as it indicates the strength and direction of the relationship between the independent variable and the probability of the dependent variable. It is suitable for dealing with binary or multiclass classification problems [39]. In addition, it is noteworthy that although the NN classifier showed the best predictive capability in the lesion level testing cohort, it did not pass the HL test, which indicates that the NN model has strong predictive ability but poor goodness-of-fit. This may be due to reasons such as imbalanced sample distribution or model overfitting [40]. This should be taken into account when using the model in clinical practice.

This study utilized SHAP to explain the influential features that contributed to the final model. At the patient level, all of the top ten influential features in the SUV/CT-based model were derived from CT, while no features from SUV was considered as the top ten influential features for the final model. In the PET/CT-based model, features from both PET and CT contributed to the model, such as pet_Idmn.4, pet_Skewness.3, and pet_Median, originating from PET, while the feature ct_SizeZoneNonUniformityNormalized.4 from CT was consistently among the top ten influential features for each type of classifier. Similar findings were found at the lesion level. Most of the top ten influential features in the SUV/CT-based model were derived from CT, with only one or two features such as suv_Min and suv_Std deviation originating from SUV. In PET/CT-based models, most of the contributed features including pet_Maximum.2, pet_ZoneEntropy.7, and pet_Complexity.5 originated from PET. Among the influential features, a higher value of ZoneEntropy indicates more heterogeneneity in the texture patterns, as it measures the uncertainty/randomness in the distribution of zone sizes and gray levels. Skewness measures the asymmetry of the distribution of values about the mean value. GrayLevelNonUniformityNormalized computes the variability of gray-level intensity values, with a lower value indicating a greater similarity in intensity values. SizeZoneNonUniformityNormalized measures the variability of size zone volumes throughout the image, with a lower value indicating more homogeneity among zone size volumes in the image [34].

Our study has certain limitations that should be considered. Firstly, our study was a retrospective analysis with a small sample from one center, which limits our model to distinguish the accurate subtypes of T-cell lymphoma, and has low accuracy. To reduce the uncertainty and instability of the models, we used cross-validation and multiple machine learning models in this study. In future studies, we need to increase the sample size and conduct multi-center validation. Secondly, when splitting the training set and test set with a ratio of 7:3 at the lesion level, we cannot make sure that all lesions from a patient are assigned to the same group, which may introduce some bias. Then, despite efforts to minimize human factors by having all segmented lesions reviewed by two experienced nuclear medicine physicians, it remains challenging to accurately segment lesions that have unclear borders on non-contrast-enhanced CT scans. These limitations should be taken into account when interpreting the results of our study.

## Conclusion

Pretreatment ^18^F-FDG PET/CT-based radiomics models accurately screen T-cell lymphoma in children, providing incremental prediction value compared to SUV-associated features. LR demonstrated the best predictive capability and goodness-of-fit at both patient and lesion levels.

### Supplementary Information

Below is the link to the electronic supplementary material.

Supplementary file1 (DOCX 17 KB)

Supplementary file2 Least absolute shrinkage and selection operator (LASSO) logistic for texture feature selection. (A) LASSO coefficient profiles of the texture features, and selection of the tuning parameter (λ) in the LASSO model in SUV/CT based model at the patient level; (B) LASSO coefficient profiles of the texture features, and selection of the tuning parameter (λ) in the LASSO model in PET/CT based model at the patient level; (C) LASSO coefficient profiles of the texture features, and selection of the tuning parameter (λ) in the LASSO model in SUV/CT based model at the lesion level; (D) LASSO coefficient profiles of the texture features, and selection of the tuning parameter (λ) in the LASSO model in PET/CT based model at the lesion level (TIF 587 KB)

Supplementary file3 Correlation matrix of CT radiomics features and PET radiomics features. (A) Correlation matrix in SUV/CT-based model at the patient level; (B) Correlation matrix in PET/CT-based model at the patient level; (C) Correlation matrix in SUV/CT-based model at the lesion level; (D) Correlation matrix in PET/CT-based model at the lesion level (TIF 898 KB)

## Data Availability

The datasets used and/or analyzed during the current study are available from the corresponding author on reasonable request.
