# Supplementary material for: Predicting T-Cell Lymphoma in Children From 18F-FDG PET-CT Imaging With Multiple Machine Learning Models
Source: J Imaging Inform Med. 2024 Feb 6;37(3):952–64. doi: 10.1007/s10278-024-01007-y (PMC11169166; doi:10.1007/s10278-024-01007-y)
Supplement: Supplementary file 1 — Supplementary file1 (DOCX 17 KB) [file 10278_2024_1007_MOESM1_ESM.docx]

Supplementary Table 1 Volume of VOI in training and validation sets.

|  |  | Patient level | | | Lesion level | | |
| --- | --- | --- | --- | --- | --- | --- | --- |
|  |  | Training set | Validation set | *P*-Value | Training set | Validation set | *P*-Value |
| Group | | | | 0.339 |  | | 0.119 |
|  | T cell | 37 | 14 |  | 107 | 56 |  |
|  | Non-T cell | 40 | 19 |  | 140 | 51 |  |
| VOI volume(cm^3^) | | | |  |  | |  |
|  | T cell | 167.802±213.271 | 141.428±150.272 | 0.682 | 5.825±37.544 | 2.355±2.134 | 0.511 |
|  | Non-T cell | 194.604±402.584 | 227.060±522.755 | 0.792 | 3.842±12.623 | 3.571±7.018 | 0.885 |

Abbreviations: VOI, volume of interest
