# Supplementary figures and images for: Predicting T-Cell Lymphoma in Children From 18F-FDG PET-CT Imaging With Multiple Machine Learning Models
Source: J Imaging Inform Med. 2024 Feb 6;37(3):952–64. doi: 10.1007/s10278-024-01007-y (PMC11169166; doi:10.1007/s10278-024-01007-y)

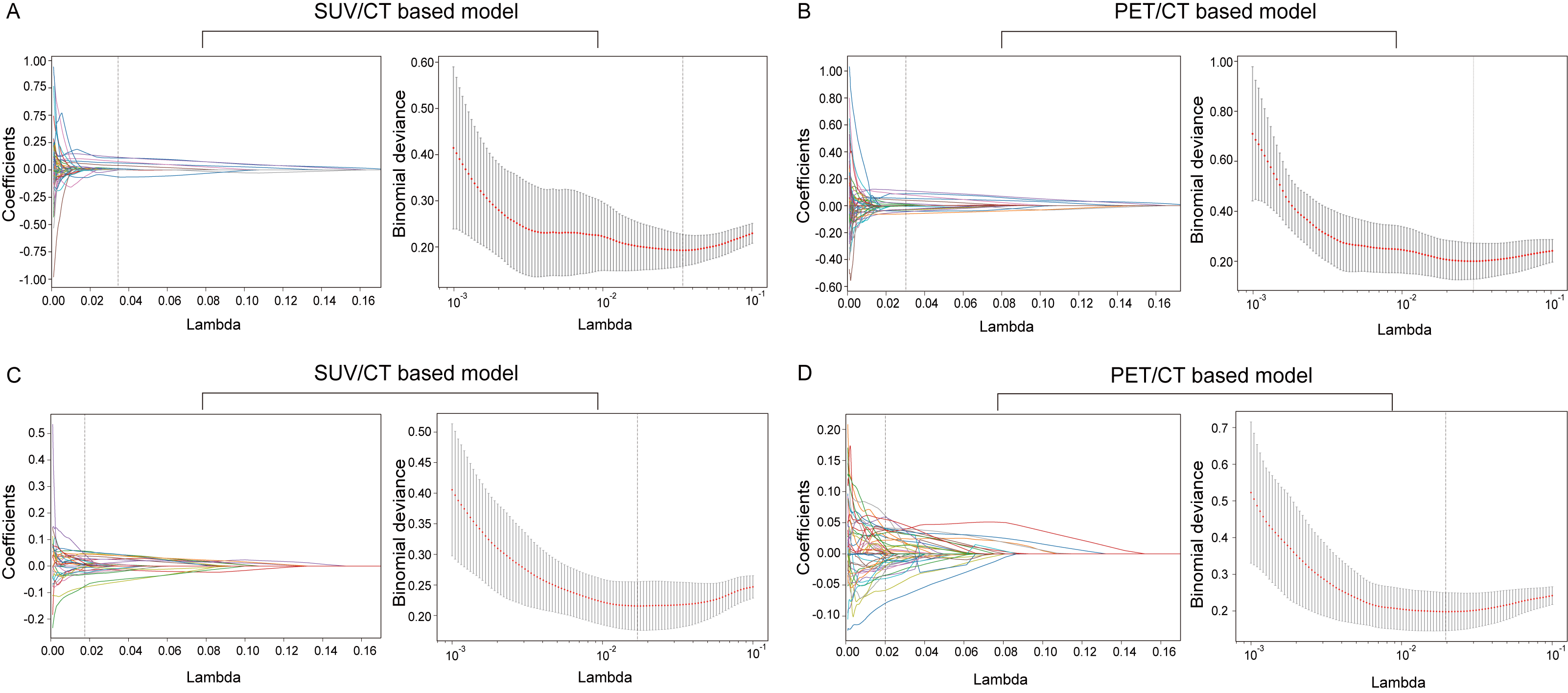

Supplement: Supplementary file 2 — Supplementary file2 Least absolute shrinkage and selection operator (LASSO) logistic for texture feature selection. (A) LASSO coefficient profiles of the texture features, and selection of the tuning parameter (λ) in the LASSO model in SUV/CT based model at the patient level; (B) LASSO coefficient profiles of the texture features, and selection of the tuning parameter (λ) in the LASSO model in PET/CT based model at the patient level; (C) LASSO coefficient profiles of the texture features, and selection of the tuning parameter (λ) in the LASSO model in SUV/CT based model at the lesion level; (D) LASSO coefficient profiles of the texture features, and selection of the tuning parameter (λ) in the LASSO model in PET/CT based model at the lesion level (TIF 587 KB) [file 10278_2024_1007_MOESM2_ESM.tif]

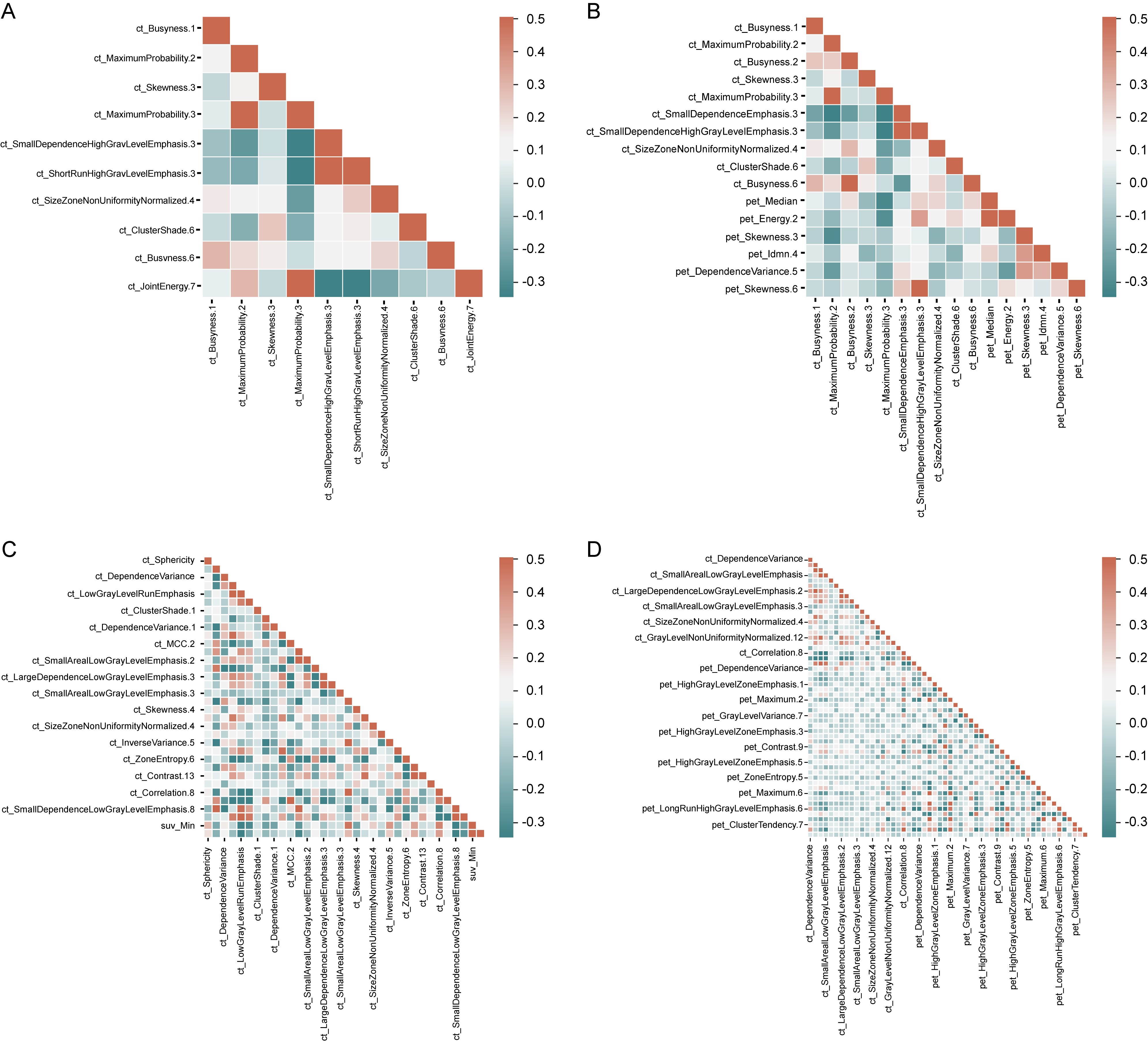

Supplement: Supplementary file 3 — Supplementary file3 Correlation matrix of CT radiomics features and PET radiomics features. (A) Correlation matrix in SUV/CT-based model at the patient level; (B) Correlation matrix in PET/CT-based model at the patient level; (C) Correlation matrix in SUV/CT-based model at the lesion level; (D) Correlation matrix in PET/CT-based model at the lesion level (TIF 898 KB) [file 10278_2024_1007_MOESM3_ESM.tif]
